# Genetic polymorphisms of *GSTM1* and *GSTT1* genes: effects on susceptibility to formaldehyde-induced hematotoxicity

**DOI:** 10.22099/mbrc.2025.54313.2213

**Published:** 2026

**Authors:** Reza Pourbabaki, Seyed Ahmadreza Siadat, Saeed Yousefinejad, Esmaeel Soleimani

**Affiliations:** 1 Student Research Committee, Shiraz University of Medical Sciences, Shiraz, Iran; 2 Department of Occupational Health and Safety Engineering, Shiraz University of Medical Sciences, Shiraz, Iran; 3 Department of Biology, School of Science, Shiraz University, Shiraz, Iran

**Keywords:** Formaldehyde, Hematotoxicity, Genetic polymorphism, Occupational exposure

## Abstract

Formaldehyde (FA) is a known human carcinogen for the upper respiratory tract. However, its hematotoxicity remains unclear. This study was performed to assess probable effects of FA on blood parameters and to determine the common polymorphisms in the detoxification enzymes *GSTM1* and *GSTT1* as biomarkers of susceptibility. Sixty-four workers with high occupational FA exposure and 57 non-exposed controls were studied. Complete blood count was performed. Also, *GSTM1*/*GSTT1* genotypes were determined. After adjusting for potential confounding variables, FA exposure was only associated with the levels of hemoglobin, mean corpuscular hemoglobin concentration, and reticulocytes. Notably, workers with homozygous deletions of *GSTM1* or *GSTT1* had hematological parameters similar to those with active genes. In conclusion, very high FA exposure resulted in only slight alterations in MCHC and no overt hematotoxicity was observed. These findings suggest that even at high exposure levels, FA may not reach the bone marrow in sufficient amounts to cause hematotoxicity. Also, it seems that *GSTM1*/*GSTT1* polymorphisms do not influence the workers’ susceptibility to FA-related blood effects.

## INTRODUCTION

Formaldehyde (FA), a ubiquitous industrial chemical, is classified as a human carcinogen by the IARC [1]. It is widely used in manufacturing resins, plastics, and disinfectants, posing significant risks in occupational settings where exposure often exceeds safety thresholds [2]. Under these conditions, individual variations in detoxification pathways may influence FA's health effects [3]. In recent years, concerns regarding FA-induced hematotoxicity and leukemia emerged [4]. The hematotoxicity of FA has been reported in both animal and human studies [5, 6]. Some studies reported the relationship between changes in blood parameters and FA exposure [5, 7]. On the other hand, some did not find such an association [8, 9]. The US Environmental Protection Agency (EPA) reported a consistent association between exposure to FA and various forms of lymphohematopoietic cancers; however, the process by which FA induces lymphohematopoietic cancers is not clearly stated [6 ,10]. On the other hand, several recent epidemiological studies did not find consistent associations between FA and hematotoxicity. These studies concluded that the current evidence does not provide convincing support for considering FA a human leukemogen [11-14]. 

Given FA’s metabolic pathway, exposure severity may play a critical role in its potential hematotoxicity. FA is highly reactive and is rapidly metabolized in the body; thus, its ability to cause distant-site toxicity (e.g., in the bone marrow) remains a matter of debate. Since the metabolic pathways of FA are present in all cells, it seems that in the case of high-level exposures, the amount of FA exceeds normal metabolic capacities which may allow FA to reach the bone marrow and subsequent blood effects [15]. 

Genetic polymorphisms in xenobiotic-metabolizing enzymes, such as glutathione S-transferases (GSTs), are key modulators of chemical detoxification. For instance, null genotypes of the *GSTM1* and *GSTT1* genes, which encode enzymes critical for conjugating glutathione to electrophilic compounds, have been linked to reduced detoxification efficiency of xenobiotics [16, 17]. FA metabolism involves the *GST-*mediated conversion to S-hydroxymethyl glutathione, eventually yielding formic acid (FO), a main metabolite of FA [18, 19]. Given the above, this study was undertaken to assess the probable hematotoxicity of FA and the effects of *GSTM1* and *GSTT1* polymorphisms, as biomarkers of susceptibility, in workers with high levels of FA exposure.

## MATERIALS AND METHODS

### Subjects and Study design:

This cross-sectional study recruited 64 individuals from a FA-producing plant who were occupationally exposed to FA. Additionally, 57 individuals, including hospital nurses and administrative staff from the FA-producing industry, were selected as the control group. None of the control participants had any occupational exposure to FA or other toxic substances such as chemotherapeutic agents. Demographic data, medical and occupational history, smoking habits, and alcohol use were documented through structured questionnaires. Blood samples were obtained from all participants for complete blood count (CBC) analysis and genotyping. Non-fasting venous blood samples (~5 mL) were collected from each participant in the morning after at least 10 minutes of seated rest. Samples were collected by standard phlebotomy into EDTA tubes and analyzed promptly using an automated hematology analyzer. The measured hematological parameters included white blood cells (WBCs), neutrophils, lymphocytes, red blood cells (RBCs), hemoglobin (Hb), hematocrit (Hct), mean corpuscular volume (MCV), mean corpuscular hemoglobin (MCH), mean corpuscular hemoglobin concentration (MCHC), platelets, platelet distribution width (PDW), reticulocytes (Ret) and lactate dehydrogenase (LDH). Prior to participation, all individuals received a detailed explanation of the study’s aims and provided an informed consent. The study was approved by the University ethic committee (IR.SUMS.SCHEANUT.REC.1402.094) and adhered to the revised guidelines of the 2000 Helsinki Declaration [20]. Exclusion criteria included smoking, alcohol consumption, pre-existing kidney or liver conditions, and any known hematological disorders (such as anemia and thalassemia) that could potentially affect hematological parameters.

### Air sampling and Analysis:

 Air sampling was conducted within each worker’s breathing zone using impingers containing 20 mL 1% sodium bisulfite solution connected to calibrated PCXR8 pumps (SKC, USA). In total, 64 full-shift personal air samples were collected at 0.5 L/min flow rate (NIOSH Method 3500). After sampling, samples were transferred to polyethylene bottles and then transferred to the laboratory. For analysis, 6 mL of 98% sulfuric acid and 0.1 mL of 1% chromotropic acid were added, followed by 15 min heating at 95°C, cooling at room temperature (2–3 h), and spectrophotometric quantification at 580 nm (Agilent Cary 60 UV-Vis) (NIOSH Method 3500) [21].

### Genotyping:

Genomic DNA was extracted from the blood samples using a commercially available kit (Qiagen DNA extraction kit) in accordance with the manufacturer’s instruction. Each DNA sample was subsequently stored at −20 °C until further analysis. The *GSTM1* and *GSTT1* genetic polymorphisms were evaluated using multiplex polymerase chain reaction (PCR) using specific primers (Table 1) followed by gel electrophoresis as described previously [22]. The β-globin gene, an internal control, was used to avoid false-negative findings. When the β-globin band was present but no PCR product was detected for *GSTM1* or *GSTT1*, the sample was classified as having a null genotype for the corresponding genes. Subjects having one or two copies of the respective gene were considered as 'positive' genotype, whereas those with homozygous deletions were classified as having the null genotype. All experiments were repeated at least twice using standard genotyping protocols to ensure accuracy. Details regarding the primer sequences and the resulting product sizes for the *GSTM1* and *GSTT1* genes are provided below.

**Table 1:** Primers used for genotyping

**Genes**	**Forward**	**Reverse**	**PCR product Size (bp)**
***GSTM1***	5’-GAACTCCCTGAAAAGCTAAAGC-3’	5’-CTTGGGCTCAAATATACGGTGG-3’	216
***GSTT1***	5’-TTCCTTACTGGTCCTCACATCTC-3’	5’-TCACCGGATCATGGCCAGCA-3’	459
**β-globin**	5’-CAACTTCATCCACGTTCACC-3’	5’-GAAGAGCCAAGGACAGGTAC-3’	267

### Statistical Analysis:

The normality of data distributions was assessed using the Kolmogorov–Smirnov test. For variables with non-normal distributions, appropriate data transformations were applied to achieve normal or near-normal distributions for subsequent analyses. Depending on the normality of each variable, either the independent-samples t-test or the Mann–Whitney U test was used for quantitative variables. Categorical variables were presented as frequencies. For blood parameters that did not follow a normal distribution, normalization was performed to determine the relationship between formaldehyde exposure and them. For this, the mean and standard deviation (SD) of the variables were calculated. Then the cumulative probability of data was obtained and the Inverse distribution functions (IDF.NORMAL. IDF.NORMAL (prob, mean, SD)) were used. This normalization method standardizes variables based on their mean and SD without logarithmic transformation. The IDF returns the value from the normal distribution, with a specified mean and SD, for which the cumulative probability is prob. All statistical analyses were performed using SPSS software (version 27), and a *p*<0.05 was considered statistically significant. A general linear regression model was employed to control for potential confounding variables. 

## RESULTS

Table 2 shows the demographic characteristics and the frequency of *GSTM1* and *GSTT1* genotypes of the participants. No statistically significant differences were observed in the mean of age, BMI, or work history between the groups. The participants were relatively young and the majority of them were males. The exposed group had slightly higher frequencies of null *GSTM1* and null *GSTT1* genotypes than the control group. The mean FA exposure level among the exposed individuals was 0.81 ± 0.59 ppm.

Table 3 displays the hematological parameters of the studied groups. Significant differences were observed in the levels of WBCs, neutrophils, lymphocytes, and platelets between the groups. However, all parameters were in their normal reference ranges. No statistically significant differences were found in other blood parameters between the groups. Also, no significant differences in hematological parameters were observed between males and females between the groups (data not shown).

**Table 2:** Demographic and occupational characteristics, and frequencies of genetic polymorphism of *GSTM1* and *GSTT1* of the studied subjects

**Variables**	**Exposed Group (n=64)** **Mean ± SD**	**Control Group (n= 57)** **Mean ± SD**	***p*** **-value**
Age (Year)	38.1 ± 7.2	36.5 ± 7.2	0.22^*^
BMI (Kg/m^2^)	25.9 ± 3.9	25.7 ± 3.9	0.81^*^
Job tenure (year)	10.0 ± 7.2	8.2 ± 5.9	0.15^*^
Daily working hours	8.00	8.00	
			
**Sex **n (%)			
Males	57 (89)	33 (57.89)	<0.001^┼^
Females	7 (11)	24 (42.10)	
Formaldehyde TWA exposure (ppm)	0.81 ± 0.59	-	NA

**Genotype Frequencies** n (%)			
*GSTM1*			
Positive genotypes (%)	31 (48.43)	29 (50.87)	0.85^┼^
Null genotype (%)	33 (51.56)	28 (49.12)	
*GSTT1*			
Positive genotypes (%)	47 (73.43)	44 (77.19)	0.39^┼^
Null genotype (%)	17 (26.56)	13 (22.80)	

^*^Independent sample t test**; **^┼ ^Chi-square test**; **TWA: time-weighted average**; **NA: Not applicable

**Table 3:** Comparison of the levels of hematological parameters in the studied groups

**Blood parameters**	**Expsed Group (n=64)** **Mean ± SD**	**Control Group (n=57)** **Mean ± SD**	***p-value***	**Normal values**
Hb (g/dl) ^*^	14.8 ± 1.65	14.5 ± 1.84	0.23	13.55-17.00
Hct (%) ^*^	45.44 ± 3.85	44.84 ± 4.92	0.41	32.00-54.00
RBC ((×10^6 ^/mm^3^) ^*^	5.23 ± 0.52	5.13 ± 0.61	0.32	4.50-5.90
WBC (×10^3 ^/mm^3^) ^**^	6.13 ± 1.54	6.77 ± 1.72	0.01^┼^	4.00-10.00
Neutrophils (%) ^**^	56.7 ± 7.78	60.14 ± 8.06	0.03^┼^	40.00-75.00
Lymphocytes (%) ^**^	39.47 ± 7.57	36.25 ± 7.98	0.02^┼^	20.00-40.00
Monocytes (%) ^**^	2.31 ± 0.75	2.13 ± 0.78	0.22	2.00-8.00-
Eosinophils (%) ^**^	1.52 ± 0.5	1.48 ± 0.50	0.79	0.00-6.00
RDW (%) ^**^	13.22 ± 1.12	13.18 ± 0.96	0.87	11.50-14.50
MPV (fL) ^*^	10.07 ± 0.79	10.18 ± 1.05	0.50	7.40-12.00
Platelets (×10^3^/mm^3^) ^**^	234.9 ± 47.08	254.9 ± 66.7	0.01^┼^	150.00-450.00
MCV (fL) ^**^	87.28 ± 6.82	87.6 ± 5.59	0.91	80.00-94.00
MCH (pg) ^**^	28.57 ± 3.1	28.38 ± 2.49	0.32	27.00-31.00
MCHC (g/dL) ^*^	32.66 ± 1.48	32.34 ± 1.29	0.17	33.00-37.00
Reticulocytes (%) ^*^	0.77 ± 0.2	0.82 ± 0.16	0.05	0.20-2.00
PDW (%) ^*^	13.27 ± 1.73	13.62 ± 2.52	0.31	9.00-17.00
LDH (U/L) ^*^	352.61 ± 60.54	341.95 ± 58.31	0.33	235.00-470.00

Hb: Hemoglobin, Hct: Hematocrit, RBC: Red blood cell, WBC: White Blood Cells, RDW: Red Cell Distribution Width, MPV: Mean Platelet Volume, MCV: Mean Corpuscular Volume, MCH: Mean corpuscular hemoglobin, MCHC: Mean Corpuscular Hemoglobin Concentration, PDW: Platelet Distribution Width, LDH: Lactate Dehydrogenase.

Table 4 shows the adjusted association between FA exposure levels and alterations in hematological parameters. After adjusting for potential confounders (sex, age, and job tenure) a significant relationship was only observed for MCHC. The regression analysis indicated that a one-unit increase in FA exposure results in 1.28-unit decrement in MCHC level.

Table 5 compares the hematological parameters of the FA-exposed workers based on the *GST* genotypes. No statistically significant differences were observed in hematological parameters among exposed subjects with different genotypes. In the *GSTM1* genotype, subjects with the null genotype had a mean exposure of 0.72 ppm (SD = 0.56), while those with the present genotype had a mean of 0.81 ppm (SD = 0.56). For the *GSTT1* genotype, the present group showed a mean exposure of 0.74 ppm (SD = 0.54), and the null group had a mean of 0.84 ppm (SD = 0.59). No statistically significant differences were observed in FA exposure between the genotype groups. Moreover, the combined genotype analysis (e.g., *GSTM1* null/*GSTT1* null, *GSTM1* present/*GSTT1* present) revealed no statistically significant associations with hematological parameters.

**Table 4:** Adjusted association between formaldehyde exposure and hematological parameters in the studied groups

**Blood parameters**	**B**	**SE**	***p-value*** ^*^
Hb	-0.62	0.41	0.13
Hct	-0.09	0.94	0.92
RBC	0.18	0.13	0.18
WBC	0.01	0.07	0.82
Neutrophils	0.99	1.82	0.58
Lymphocytes	-2.29	1.99	0.25
Monocytes	0.09	0.17	0.58
Eosinophils	0.04	0.09	0.62
RDW	-0.04	0.32	0.90
MPV	-0.12	0.23	0.60
Platelets	1.94	2.68	0.87
MCV	-0.73	1.90	0.70
MCH	-1.03	0.89	0.25
MCHC	-1.28	0.39	<0.01
Reticulocytes	0.01	0.06	0.81
PDW	0.58	-0.15	0.11
LDH	11.99	17.96	0.50

Hb: Hemoglobin, Hct: Hematocrit, RBC: Red blood cell, WBC: White Blood Cells, RDW: Red Cell Distribution Width, MPV: Mean Platelet Volume, MCV: Mean Corpuscular Volume, MCH: Mean corpuscular hemoglobin, MCHC: Mean Corpuscular Hemoglobin Concentration, PDW: Platelet Distribution Width, LDH: Lactate Dehydrogenase.

**Table 5:** Comparison of the means of hematological parameters between *GSTM1* and *GSTT1* genotypes in the exposed group

**Blood parameters**	***GSTM1 *** **genotypes** **Mean ± SD**		***p-value***		***GSTT1*** ** genotypes** **Mean ± SD**		***p-value***
	**Positive** **(n=31)**	**Null** **(n=33)**			**Positive** **(n=47)**	**Null** **(n=17)**	
Hb (g/dl) *	14.87 ± 1.85	14.86 ± 1.47	0.98		14.97 ± 1.74	14.57 ± 1.35	0.40
Hct (%) *	45.50 ± 4.47	45.38 ± 3.23	0.90		45.64 ± 4.02	44.88 ± 3.38	0.48
RBC ((×10^6^ /mm^3^) *	5.23 ± 0.55	5.22 ± 0.51	0.94		5.22 ± 0.54	5.25 ± 0.48	0.84
WBC (×10^3^ /mm^3^) **	5.61 ± 1.20	6.30 ± 1.70	0.62		5.00 ± 1.2	6.50 ± 2.15	0.36
Neutrophils (%) **	56.14 ± 7.32	56.13 ± 7.32	0.62		57.47 ±7.12	54.59 ± 9.57	0.14
Lymphocytes (%) **	40.13 ± 6.89	38.85 ± 8.22	0.57		38.91 ± 6.94	41.00 ± 9.17	0.21
Monocytes (%) **	2.32 ± 0.65	2.30 ± 0.84	0.82		2.19 ± 0.77	2.65 ± 0.60	0.08
Eosinophils (%) **	1.42 ± 0.50	1.61 ± 0.49	0.13		1.43 ± 0.50	1.76 ± 0.43	0.06
RDW (%) **	13.20 ± 1.01	13.25 ± 1.23	0.97		13.10 ± 0.92	13.55 ± 1.54	0.29
MPV (fL) *	9.96 ± 0.78	10.17 ± 0.80	0.29		10.06 ± 0.75	10.08 ± 0.92	0.91
Platelets (×10^3^/mm^3^) **	240.7 ± 53.8	229.4 ± 39.9	0.38		233.85 ± 49.90	237.76 ± 39.3	0.67
MCV (fL) **	87.20 ± 6.42	87.34 ± 7.28	0.85		87.79 ± 6.63	85.87 ± 7.36	0.28
MCH (pg) **	28.51 ± 3.04	28.62 ± 3.21	0.69		28.79 ± 3.02	27.94 ± 3.34	0.32
MCHC (g/dL) *	32.62 ± 1.54	32.70 ± 1.45	0.83		32.73 ± 1.47	32.47 ± 1.54	0.54
Reticulocytes (%) **	0.75 ± 0.18	0.79 ± 0.23	0.64		0.76 ± 0.20	0.80 ± 0.23	0.55
PDW (%) *	12.95 ± 1.76	13.47 ± 1.69	0.23		13.24 ± 1.73	13.17 ± 1.78	0.89
LDH (U/L) *	344.94 ± 58.5	359.82 ± 62.3	0.33		354.53 ± 59.2	347.29 ± 65.5	0.67

^*^^‎^Independent Samples T-tes; ^**^Mann-Whitney U test; *p*<0.05 statistically significant

Hb: Hemoglobin, Hct: Hematocrit, RBC: Red blood cell, WBC: White Blood Cells, RDW: Red Cell Distribution Width, MPV: Mean Platelet Volume, MCV: Mean Corpuscular Volume, MCH: Mean corpuscular hemoglobin, MCHC: Mean Corpuscular Hemoglobin Concentration, PDW: Platelet Distribution Width, LDH: Lactate Dehydrogenase.

## DISCUSSION

In the present study, the effects of *GSTM1* and *GSTT1* polymorphisms, as biomarkers of susceptibility, on the probable hematotoxicity of FA in workers with high exposures to FA were investigated. In an experimental study, Zhang et al. (2010) for the first time reported that FA exposure may result in hematological changes and leukemia [7]. On the other hand, epidemiological studies have yielded inconsistent results. For instance, some studies reported significant changes in at least one blood parameter in FA-exposed workers compared to controls [7, 23]. In contrast, others found no association between FA exposure and blood dyscrasia [8, 24]. It is noteworthy that some studies reported blood effects even at low exposure levels (0.02 to 0.08 ppm) [23, 26]. While others with higher levels did not find significant effects [8, 28]. While there are some methodological flaws in the FA exposure assessment in some of these studies, the main reason for inconsistent findings in hematotoxicity of FA can be found in its toxicokinetics. In the body, FA is rapidly metabolized and cannot induce toxicity on distant tissues (e.g., to bone marrow). However, in high exposure severities, the amount of FA exceeds the normal metabolic capacities which may result in reaching exogenous FA to the bone marrow [15]. Meanwhile, the polymorphisms in the *GSTM1* and *GSTT1* genes are critical to FA metabolism. In individuals with null genotypes, higher levels of FA may reach distant tissues, including bone marrow. 

 In the present study, workers were exposed to high levels of FA. The TWA exposure of the exposed workers was 0.81 ± 0.59 ppm, about eight times higher than its current TLV-TWA of 0.1 ppm [28]. After adjusting for potential confounders, significant relationship was only observed for MCHC (B = -1.28, *p*<0.01). No significant relationship was observed for other parameters, especially WBC and its subsets. These findings are in line with those reported by other authors [5, 29, 30]. For example, in one study with a mean exposure concentration of 0.1 ppm, no changes in hematological parameters were observed despite the higher exposure levels [8]. We cannot directly attribute this change in MCHC to FA exposure. Some studies showed that body fat content is a risk factor for RBC-related parameters. Animal studies reported RBC dysfunction and reduced its survival in obese rats [31]. In addition, an increasing body of evidence suggests an association between blood cholesterol/triglycerides and hematological diseases [32]. Also, higher levels of RDW and Hb have been reported in non-alcoholic fatty liver patients [33, 34]. The exposed group had a mean BMI of 25.90±3.90 kg/m^2^ and 42 and 15.6 % of them were overweight and obese, respectively. Therefore, reduced levels of the MCHC (Table 4) may be because of high body fat content, cholesterol and triglycerides levels. 

The genetic polymorphisms of the biotransformation enzymes are an important example of biomarkers of susceptibility that predispose some individuals to toxic effects of some chemicals [35]. In the present study, 24.79 and 50.41% of the workers had null *GSTT1* and *GSTM1*, respectively, which is similar to those found in different Iranian populations [36]. No significant differences were observed in the frequencies of null *GSTM1* and *GSTT1* between the groups. The severity of exposure to FA between the genotype groups was not significantly different. Therefore, it is expected that in the null genotype workers, FA is metabolized less efficiently, leading to higher blood levels of FA and probably subsequent toxicity. To address this, we compared the hematological parameters between workers with different *GSTT1* and *GSTM1* genotypes. None of the blood parameters were significantly different between workers with different *GSTT1* and *GSTM1* genotypes (Table 5). These findings along with the relatively long duration of exposure (10.0 ± 7.2 years), tentatively support the notion that FA cannot exert distant site toxicity, even at high exposure severities. 

Some major strengths of the present study are as follows. First, we performed an extensive exposure monitoring program and provided a good estimation of workers’ exposure to FA. Second, a wide range of exposure severity and duration was studied. Third, none of the exposed and non-exposed subjects were smokers, none consumed alcohol, none were exposed to any other chemicals known to blood toxicity, and none had preexisting medical conditions affecting the hematopoietic system. Forth, we, for the first time, assessed the effects of the *GSTM1* and *GSTT1* genes polymorphisms on the possible FA blood toxicity. However, the study had some limitations such as a small sample size (64 exposed and 57 non-exposed subjects), a narrow focus on *GSTM1* and *GSTT1* polymorphisms, without exploring other FA detoxification pathways that may overlook compensatory metabolic mechanisms and the exclusion of polymorphisms in other FA-metabolizing genes that restrict the scope of genetic susceptibility analysis. 

### Acknowledgements:

The present study was a part of the results of the PhD thesis of the first author, Reza Pourbabaki, supervised by Dr. Soleimani, the paper’s last author. The authors would like to appreciate the financial support provided by the Shiraz University of Medical Sciences. The authors would like to thank the employers and employees for their cooperation in the study. We also thank Dr. Rezvan Zendehdel for providing some chemicals used in the study. 

### Conflict of Interest:

The authors declare that they have no conflict of interest.

### Authors’ Contribution:

 ES: Conceptualization, Funding acquisition, Methodology, Project administration, Resources, Supervision. RP: Data curation, Investigation, Validation, Writing–original draft, Methodology. SAS: Methodology, Visualization. SY: Methodology, Validation. 

### Ethics approval:

Informed consent was obtained from all individual participants included in the study.

### Consent to participate:

Informed consent was obtained from all individual participants included in the study.
